# A High Accuracy Voltage Approximation Model Based on Object-oriented Sensitivity Matrix Estimation (OO-SME Model) in Electrical Impedance Tomography

**DOI:** 10.2478/joeb-2022-0015

**Published:** 2023-01-08

**Authors:** Zengfeng Gao, Panji Nursetia Darma, Daisuke Kawashima, Masahiro Takei

**Affiliations:** 1 Division of Fundamental Engineering, Graduate School of Science and Engineering, Chiba University, Chiba, Japan

**Keywords:** Electrical impedance tomography, object-oriented sensitivity matrix estimation, high reconstruction accuracy

## Abstract

The image reconstruction in electrical impedance tomography (EIT) has low accuracy due to the approximation error between the measured voltage change and the approximated voltage change, from which the object cannot be accurately reconstructed and quantitatively evaluated. A voltage approximation model based on object-oriented sensitivity matrix estimation (OO-SME model) is proposed to reconstruct the image with high accuracy. In the OO-SME model, a sensitivity matrix of the object-field is estimated, and the sensitivity matrix change from the background-field to the object-field is estimated to optimize the approximated voltage change, from which the approximation error is eliminated to improve the reconstruction accuracy. Against the existing linear and nonlinear models, the approximation error in the OO-SME model is eliminated, thus an image with higher accuracy is reconstructed. The simulation shows that the OO-SME model reconstructs a more accurate image than the existing models for quantitative evaluation. The relative accuracy (RA) of reconstructed conductivity is increased up to 83.98% on average. The experiment of lean meat mass evaluation shows that the RA of lean meat mass is increased from 7.70% with the linear model to 54.60% with the OO-SME model. It is concluded that the OO-SME model reconstructs a more accurate image to evaluate the object quantitatively than the existing models.

## Introduction

Electrical impedance tomography (EIT) is a promising visualization technique with low hardware cost, which reconstructs the conductivity change inside a domain from the voltage change on the boundary [1]. EIT has the potential to evaluate the effect of electrical muscle stimulation (EMS) on human muscles [2,3], where EMS is being expected to replace physical exercises of human body in the future. However, the EIT image has low accuracy [4], which restricts the accurate reconstruction of muscle compartments and the quantitative evaluation of EMS effect [5,6]. To increase the reliability of quantitative evaluation of EMS, the reconstruction accuracy of muscle compartments needs to be improved.

EIT reconstructs the image by matching the measured voltage change Δ**U** with an approximated voltage change ***u***(Δ**σ**) resulting from the conductivity change Δ**σ**, in which the approximation error ***e*** between Δ**U** and ***u***(Δ**σ**) causes the low accuracy of image reconstruction. Generally, ***u***(Δ**σ**) is formulated as the product of a sensitivity matrix and the conductivity change based on a linear model [7]. With conductivity **σ** changing from the background-field **σ***^b^* to the object-field **σ***^o^* = **σ***^b^*+Δ**σ**, the voltage **U** changes from **U***^b^* to **U***^o^* = **U***^b^*+Δ**U** accordingly, and the nonlinear function **U** = ***f***(**σ**) mapping **σ** to **U** changes from ***f****^b^*(**σ***^b^*) to ***f****^o^*(**σ***^o^*). Replacing operator ***f****^o^* with ***f****^b^* and using the Taylor formula on ***f****^b^*(**σ***^o^*) the result ***u***(Δ**σ**) is formulated as **J***^b^*Δ**σ** [8], where **J***^b^* is the Jacobian matrix of ***f****^b^*(**σ***^b^*). For simplicity, **J***^b^* is replaced by the sensitivity matrix **S***^b^*, which is calculated from a linearized form of ***f****^b^*(**σ***^b^*) [9]. As ***e*** between Δ**U** and ***u***(Δ**σ**) is non-negligible, the reconstructed conductivity change Δσ^*^ has low accuracy, which influences the further quantitative evaluation [4].

To reduce ***e*** between Δ**U** and ***u***(Δ**σ**), two nonlinear models were modified from the linear model to approximate ***u***(Δ**σ**) by optimizing **S***^b^*. The first nonlinear model is the so-called “sensitivity updating model” [10], in which **S***^b^* is updated. In detail, a conductivity **σ**^b^^*^ as the new background-field is used to calculate the new sensitivity matrix **S**^b^^*^. The second nonlinear model is the so-called “second-order sensitivity model” [11], in which the Hessian matrix of ***f****^b^*(**σ***^b^*) is estimated as **S***^b^*^†^ to compensate for **S***^b^*. In detail, the *m*^th^ row of **S***^b^*^†^ is represented by the pivots of [**S***^b^*]*_m_^T^*[**S***^b^*]*_m_*, where [**S***^b^*]*_m_* is the *m*^th^ row of **S***^b^*. However, the reduction of ***e*** by optimizing **S***^b^* in the two nonlinear models is insufficient. Analytically, with **σ** changing from **σ***^b^* to **σ***^o^*, the operator ***f*** changes from ***f****^b^* to ***f****^o^* accordingly. The change of ***f*** has an inevitable influence on ***u***(Δ**σ**), in which the influence reflects on **σ***^b^* and Δ**σ** simultaneously. To eliminate ***e*** thoroughly, it is necessary to consider the influence of change of ***f*** on ***u***(Δ**σ**) completely.

Under these circumstances, a new voltage approximation model for conductivity reconstruction is proposed, which is called the “object-oriented sensitivity matrix estimation model (OO-SME model)”. The OO-SME model is derived by linearizing **U** = ***f***(**σ**) as the product of a sensitivity matrix **S** and the conductivity **σ**. Thus, two linear equations are formulated simultaneously on **σ***^b^* as **U***^b^* = **S***^b^***σ***^b^* and on **σ***^o^* as **U***^o^* = **S***^o^***σ***^o^*, where **S***^b^* and **S***^o^* are the sensitivity matrices corresponding to **σ***^b^* and **σ***^o^* respectively. Therefore, ***u***(Δ**σ**) resulting from Δ**σ** is related to **σ***^b^*, Δ**σ**, **S***^b^*, and Δ**S** in the OO-SME model, where Δ**S** is the sensitivity matrix change from **S***^b^* to **S***^o^*. In the OO-SME model, ***e*** between Δ**U** and ***u***(Δ**σ**) is eliminated, thus, an image with higher accuracy can be reconstructed.

The objectives of this study are (1) to propose the OO-SME model for conductivity reconstruction with high accuracy, (2) to reconstruct the lean meat in meat sample accurately as a mimic reconstruction of muscle compartment, and (3) to evaluate the mass of lean meat quantitatively from the reconstruction.

## Conductivity reconstruction with OO-SME model

### OO-SME model for voltage change approximation based on object-oriented sensitivity matrix estimation

The approximation error ***e*** between approximated voltage change ***u***(Δ**σ**) and measured voltage change Δ**U** is (1),


(1) e=ΔU−u(Δσ)

where Δ**U** is the measured voltage change from background-field with conductivity **σ***^b^* to object-field with conductivity **σ***^o^* = **σ***^b^*+Δ**σ**, ***u***(Δ**σ**) is the approximated voltage change resulting from the conductivity change Δ**σ**. In the OO-SME model, ***u***(Δ**σ**) is approximated as (2),


(2) $$u(\text{Δ}\sigma )=S^{b}\text{Δ}\sigma +\text{Δ}S\sigma^{b}+\text{Δ}S\text{Δ}\sigma$$

where **S***^b^* is the sensitivity matrix calculated from **σ***^b^*, Δ**S** is the sensitivity matrix change from **S***^b^* to **S***^o^*, where **S***^o^* is the sensitivity matrix calculated from **σ***^o^*. **S***^b^* and **S***^o^* are calculated by (3a) and (3b),


(3a) $${\left[S^{b}\right]}_{m,e}=\frac{1}{I}\int_{{\text{Ω}}_{e}}\left[\nabla \varphi_{m-c}\left(\sigma^{b}\right)\cdot \nabla \varphi_{m-v}\left(\sigma^{b}\right)\right]{\text{dΩ}}_{e}$$

(3b) $${\left[S^{o}\right]}_{m,e}=\frac{1}{I}\int_{{\text{Ω}}_{e}}\left[\nabla \varphi_{m-c}\left(\sigma^{o}\right)\cdot \nabla \varphi_{m-v}\left(\sigma^{o}\right)\right]{\text{dΩ}}_{e}$$

where *e* is the element index, Ω*_e_* is the pixel of *e*^th^ element, *φ_m_*_-*c*_(•) and *φ_m_*_-*v*_(•) are the field potentials relevant to *m*^th^ electrode-combination, *m*-*c* and *m*-*v* represent the current stimulating with current-stimulation pair and voltage-measurement pair respectively [9]. *I* is the current amplitude. Δ**S** is calculated from **S***^b^* and **S***^o^* by (4),


(4) $$\text{Δ}S=S^{o}-S^{b}$$

and used to optimize ***u***(Δ**σ**) by (2). In practice, an estimated conductivity **σ**^o^^*^ of the object-field is used to calculate the estimated sensitivity matrix **S**^o^^*^, from which an estimated sensitivity matrix change ΔS^*^ is calculated to replace Δ**S**.

The OO-SME model is derived by linearizing **U** = ***f***(**σ**) as a product of the sensitivity matrix **S** and the conductivity **σ** as (5) based on the divergence theorem [9],


(5) $${\left[U\right]}_{M\times 1}={\left[S\right]}_{M\times N}{\left[\sigma \right]}_{N\times 1}$$

where **U** is the voltage from the boundary with different electrode-combination, **σ** is the discretized conductivity in the domain, **S** is the sensitivity matrix. *M* is the total number of electrode-combinations, *N* is the total number of elements. Based on (5), the voltage **U***^b^* from the conductivity **σ***^b^* is formulated as (6-a),


(6-a) $${\left[U^{b}\right]}_{M\times 1}={\left[S^{b}\right]}_{M\times N}{\left[\sigma^{b}\right]}_{N\times 1}$$

the voltage **U***^o^* from the conductivity **σ***^o^* is formulated as (6-b).


(6-b) $${\left[U^{o}\right]}_{M\times 1}={\left[S^{o}\right]}_{M\times N}{\left[\sigma^{o}\right]}_{N\times 1}$$

Replacing **U***^o^* with **U***^b^*+Δ**U**, **S***^o^* with **S***^b^*+Δ**S**, and **σ***^o^* with **σ***^b^*+Δ**σ** in (6-b) and subtracting (6-a) from (6-b) gets the expression of Δ**U** from **U***^b^* to **U***^o^*, which is used to formulate ***u***(Δ**σ**) in the OO-SME model in (2).

Compared to the existing linear and nonlinear models, ***u***(Δ**σ**) in the OO-SME model is optimized by considering the influence of Δ**S** completely. As a result, ***e*** between ***u***(Δ**σ**) and Δ**U** is significantly eliminated. Thus, Δ**σ** is expected to be reconstructed with a higher accuracy.

### Conductivity reconstruction with OO-SME model

**Fig. 1:**
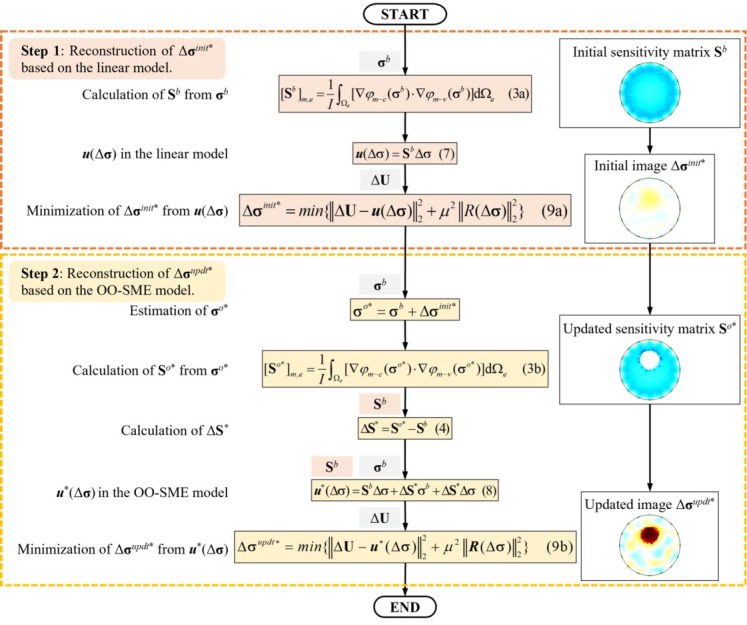
Flowchart of conductivity reconstruction with the OO-SME model.

Fig. 1 shows the flowchart of conductivity reconstruction with the OO-SME model, which includes two steps. In the first step, an initial conductivity change Δ**σ**^init^^*^ is reconstructed from **S***^b^* based on the linear model. In the second step, an updated conductivity change Δ**σ**^updt^^*^ is reconstructed from **S**^o^^*^ based on the OO-SME model.

In the 1^st^ step of reconstructing Δ**σ**^init^^*^, **S***^b^* is calculated from **σ***^b^* by (3a), ***u***(Δ**σ**) is formulated based on the linear model as (7).


(7) $$u(\text{Δ}\sigma )=S^{b}\text{Δ}\sigma$$

Δ**σ**^init^^*^ is reconstructed by matching Δ**U** with ***u***(Δ**σ**).

In the 2^nd^ step of reconstructing Δ**σ**^updt^^*^, the process includes three parts. Firstly, estimate the conductivity of object-field as **σ**^o^^*^ = **σ***^b^* + Δ**σ**^init^^*^, and calculate the estimated sensitivity matrix **S**^o^^*^ from **σ**^o^^*^ by (3b). Secondly, calculate the estimated sensitivity matrix change ΔS^*^ by (4), and formulate u^*^(Δ**σ**) based on the OO-SME model as (8).


(8) $$u^{*}(\text{Δ}\sigma )=S^{b}\text{Δ}\sigma +\text{Δ}S^{*}\sigma^{b}+\text{Δ}S^{*}\text{Δ}\sigma$$

Thirdly, reconstruct Δ**σ**^updt^^*^ by matching Δ**U** with u^*^(Δ**σ**). By using the OO-SME model, Δ**σ**^updt^^*^ is output with a higher accuracy than Δ**σ**^init^^*^.

To stabilize the ill-posed conductivity reconstruction model, the matching between Δ**U** with ***u***(Δ**σ**) and u^*^(Δ**σ**) are replaced by minimizing a regularized least square error function as (9a) and (9b) respectively [12],


(9a) $$\text{Δ}\sigma^{ini{*}^{*}}=min\left\{∥\text{Δ}U-u(\text{Δ}\sigma ){∥}_{2}^{2}+\mu^{2}∥R(\text{Δ}\sigma ){∥}_{2}^{2}\right\}$$

(9b) $$\text{Δ}\sigma^{updt*}=min\left\{\text{Δ}U-u^{*}{\left(\text{Δ}\sigma \right)}_{2}^{2}+\mu^{2}∥R(\text{Δ}\sigma ){∥}_{2}^{2}\right\}$$

where ‖*R*(Δ**σ**)‖ is the regularization term, and *μ* is the regularization factor. Iterative methods such as the steepest descent method (SDM) [13], the Gauss-Newton method (GN) [14], and the conjugate gradient method (CG) [15] have been used to minimize (9a) and (9b). In this study, CG is used because of its lower computational cost and the faster convergence speed.

## Conductivity reconstruction by simulation

### Voltages and sensitivity matrices of background-field and object-field

**Fig. 2:**
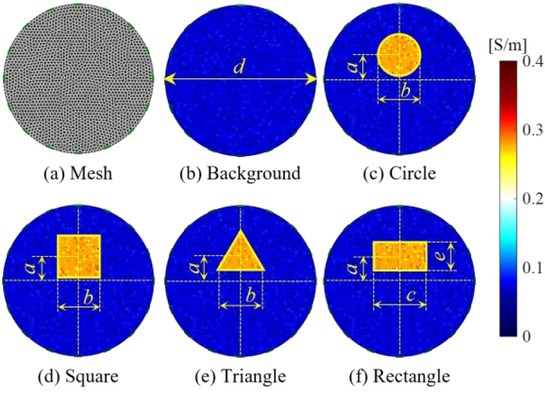
Mesh, conductivity of background- and object-fields

Fig. 2 shows a mesh, the conductivity of the background-field, and the object-fields in the simulation. Fig. 2(a) is a 2D mesh with the following parameters: diameter *d* = 100 mm, nodes number *P* = 1958, elements number *N* = 3767, electrodes number *L* = 16. Fig. 2(b) is a background-field with conductivity **σ***^b^*. Fig. 2(c) – (f) are four object-fields with conductivity **σ***^o^* = **σ***^b^* + Δ**σ**. The positions of objects are defined by parameters *a*, *b*, *c*, *d*, and *e*, where *a* = 0.32*d*, *b* = 0.27*d*, *c* = 0.34*d*, and *e* = 0.18*d*. The magnitude of **σ***^b^* and **σ***^o^* are 0.021 S/m and 0.267 S/m, respectively, thus, the magnitude of Δ**σ** is 0.246 S/m. In the simulation, a random conductivity noise δ**σ** with a magnitude of 20% of Δ**σ** is added to a partial of 40% of the elements to approximate the inconsistency of conductivity. The electrode-combination for current-stimulation and voltage-measurement is chosen as quasi-adjacent pattern [16] because of its higher signal-to-noise ratio (*SNR*) than the adjacent pattern [17]. The amplitude of current stimulation is *I* = 1 mA.

To obtain the voltages **U***^b^* and **U***^o^*, and the sensitivity matrices **S***^b^* and **S***^o^* of the background-field and object-field in the simulation, an elliptical partial differential equation with Neumann boundary is solved by the finite element method (FEM) to calculate the potential distribution [18]. The governing equation of quasi-static electric field and boundary condition of current stimulation are expressed by (10) and (11) [19],


(10) ∇⋅[σ∇φ(σ)]=0

(11) $$\{\begin{array}{c} \int_{\text{Γ}}\sigma \frac{\partial \varphi }{\partial n}\cdot \text{dΓ}=I\text{ Γ}\in E_{l}\;l=1,2,\cdots L\\ {\frac{\partial \varphi }{\partial n}|}_{\text{Γ}}=0\text{ Γ}\in \text{Γ}/\overset{L}{\underset{l=1}{\cup }}E_{l} \end{array}$$

where *σ* is the conductivity, *φ*(*σ*) is the field potential, Ω is the field domain, Γ is the boundary of Ω, d**Γ** is the outwards area element vector, ***n*** is the outwards normal vector on Γ, *I* is the current amplitude, and *E_l_* is the boundary of the *l*^th^ electrode.

The solution of *φ*(*σ*) corresponding to the discretized background-field and object-field are represented by *φ*(**σ***^b^*) and *φ*(**σ***^o^*). **U***^b^* and **U***^o^* are extracted from *φ*(**σ***^b^*) and *φ*(**σ***^o^*), which are the column vectors with size *M*×1. **S***^b^* and **S***^o^* are calculated from *φ*(**σ***^b^*) and *φ*(**σ***^o^*), which are two matrices with size *M*×*N*.

### Regularization matrix and regularization factor

Regularization is used in (9a) and (9b) to stabilize the conductivity reconstruction [20], in which regularization matrix ‖*R*(Δ**σ**)‖ and regularization factor *μ* are needed.

Different ‖*R*(Δ**σ**)‖ provides different form of penalty. The Tikhonov [21] and Noser [22] regularizations are suggested to penalize the finiteness of conductivity on individual element. The Laplace [23] and total variation (TV) [24] regularizations are suggested to consider the continuity of conductivity on adjacent elements. A hybrid regularization is used to balance the finiteness and continuity simultaneously [25]. In this study, a hybrid regularization with Noser and Laplace is used. The regularization term is calculated as (12).


(12) $$∥R(\sigma ){∥}_{2}^{2}=0.5{\left\|R_{\text{Noser }}(\sigma )\right\|}_{2}^{2}+0.5{\left\|R_{\text{Laplace }}(\sigma )\right\|}_{2}^{2}$$

The Noser regularization term of **σ** is calculated by (13),


(13) $${\left\|R_{\text{Noser }}(\sigma )\right\|}_{2}^{2}=\sum_{e=1}^{N}\omega_{e}\sigma_{e}^{2}$$

where *ω_e_* is the square sum of *e*^th^ column of **S***^b^*, and **σ***_e_* is the *e*^th^ element of **σ**. The Laplace regularization term of **σ** is calculated by (14),


(14) $${\left\|R_{\text{Laplace }}(\sigma )\right\|}_{2}^{2}=\sum_{e=1}^{N}\left[\sigma_{e}\left(\sigma_{e}-\sigma_{l}\right)+\sigma_{e}\left(\sigma_{e}-\sigma_{m}\right)+\sigma_{e}\left(\sigma_{e}-\sigma_{n}\right)\right]$$

where *l*, *m*, *n*, and *e* are element indices, *l*^th^, *m*^th^, and *n*^th^ elements are adjacent to the *e*^th^ element.

Regularization factor *μ* plays crucial role in stabilizing the conductivity reconstruction from noisy voltage ΔU^*^ due to the condition number of the sensitivity matrix is high [26], where the influence of noise is inhibited by choosing *μ* properly [27]. In this study, a Gaussian white noise δ**U***^b^* is added to Δ**U** to generate ΔU^*^ as (15),


(15) $$\text{Δ}U^{*}=\text{Δ}U+\delta U^{b}$$

where δ**U***^b^* is quantified by **U***^b^* from the simulation and *SNR* from the experiment. *SNR* is defined as (16),


(16) $$SNR=avg\left(20\log_{10}\frac{U^{b^{*}}}{\delta U^{b^{*}}}\right)$$

where **U**^b^^*^ is the voltage from the experiment, δ**U**^b^^*^ is the estimated noise from **U**^b^^*^ based on reciprocity. The magnitude of δ**U***^b^* is controlled by (17).


(17) $$\max\left\{\delta U^{b}\right\}=10^{-(SNR/20)}\max\left\{U^{b}\right\}$$

*μ* is analyzed by the truncated singular value decomposition (TSVD) as (18) [28],


(18) $$\text{Δ}\sigma^{*}=\sum_{k=1}^{K}\frac{v_{k}u_{k}^{T}}{\sigma_{k}}{\left[S^{b}\right]}^{T}\text{Δ}U+\sum_{k=1}^{K}\frac{v_{k}u_{k}^{T}}{\sigma_{k}}{\left[S^{b}\right]}^{T}\delta U^{b}$$

where Δσ^*^ is the reconstruction of Δ**σ**, *σ_k_* is the *k*^th^ singular value of **S***^b^*, **u***_k_* and **v***_k_* are the left and right orthonormal vectors of **S***^b^* corresponding to *σ_k_*, and *K* is the rank of **S***^b^*. Δ**U** is the voltage change from **U***^b^* to **U***^o^*, δ**U***^b^* is a perturbation of **U***^b^*. To ensure stable reconstruction, *μ* is chosen by guaranteeing the second term is smaller than the first term in (18). Thus, *μ* for stable reconstruction satisfies (19),


(19) $$\frac{\sigma_{1}}{\mu^{2}}\leq \max\left\{\frac{U^{b}}{\delta U^{b}}\right\}\approx 10^{\left(SNR/20\right)}$$

where *σ*_1_ is the maximum singular value of **S***^b^*.

### Voltage approximation

***u***(Δ**σ**) is different in different conductivity reconstruction models. ***u***(Δ**σ**) in the linear model is formulated by (7). In the sensitivity updating model, ***u***(Δ**σ**) is updated by an updated sensitivity matrix **S**^b^^*^ that is calculated from a new background-field **σ**^b^^*^, which is formulated as (20).


(20) $$u^{updt}(\text{Δ}\sigma )=S^{b*}\text{Δ}\sigma$$

In the second-order sensitivity model, ***u***(Δ**σ**) is compensated by an estimated second-order sensitivity matrix **S***^b^*^†^, which is formulated as (21).


(21) $$u^{comp\;}(\text{Δ}\sigma )=\left(S^{b}+S^{b†}\right)\text{Δ}\sigma$$

In the OO-SME model, ***u***(Δ**σ**) is approximated based on (2), in practice, it is formulated as (8). In the forthcoming subsection, the influence of ***u***(Δ**σ**) among the existing models and the OO-SME model will be compared by Δσ^*^.

### Evaluation of reconstructed conductivity

The relative accuracy (*RA*) between Δσ^*^ and Δ**σ** is defined to verify the high accuracy of the OO-SME model for conductivity reconstruction. *RA* is defined as (22),


(22) $$RA=\frac{1}{N^{o}}\sum_{i=1}^{N^{o}}\left|\text{Δ}\sigma_{i}^{*}-\text{Δ}\sigma_{i}\right|/\left|\text{Δ}\sigma_{i}\right|$$

where *Δ*σ^*^ *_i_*and Δ**σ***_i_* are the *i*^th^ component of Δσ^*^ and Δ**σ** respectively as well belong to the object.

### Simulation result

**Fig. 3:**
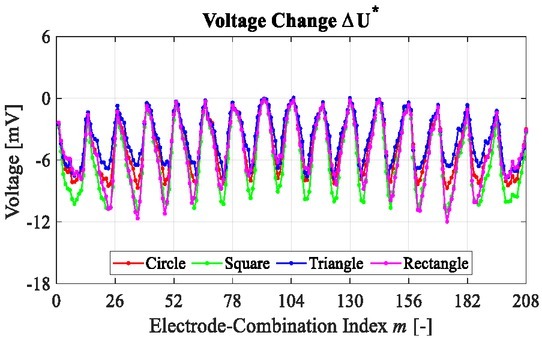
Voltage changes of different objects in the simulation.

Fig. 3 shows the voltage change ΔU^*^ of different objects in the simulation. ΔU^*^ is divided into 16 loops, and in each loop it has 13 measurements, where the value varies periodically with the electrode-combinations. Fig. 3 indicates that the conductivity change in the domain is reflected reliably by the voltage change on the boundary.

**Fig. 4:**
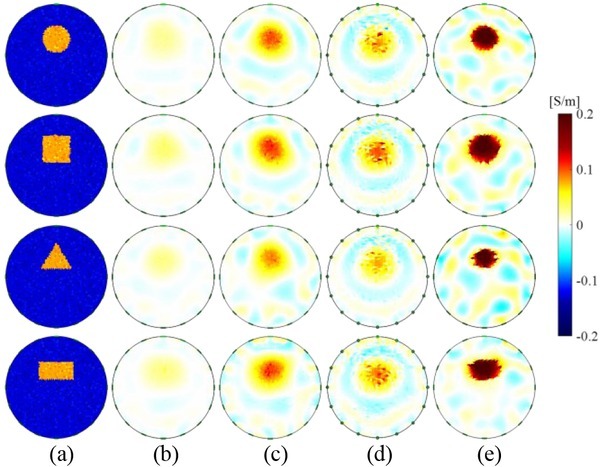
Reconstructed conductivity based on different conductivity reconstruction models in the simulation. (a) Object-fields; (b) Linear model; (c) Sensitivity updating model; (d) Second-order sensitivity model; (e) OO-SME model.

Fig. 4 shows the reconstructed conductivity Δσ^*^ based on the linear model, two nonlinear models, and the OO-SME model in the simulation. Fig. 4(a) shows four object-fields with different objects. Fig. 4(b), (c), and (d) show Δσ^*^ based on the linear model, sensitivity updating model, and second-order sensitivity model, respectively. Fig. 4(e) shows *Δ*σ^*^ based on the OO-SME model. Compared to the objects in Fig. 4(a), *Δ*σ^*^ in Fig. 4(b), (c), and (d) displays the position of the object only, in which the quantitative values of *Δ*σ^*^ are not reconstructed accurately. In contrast, besides the accurate position information, the magnitude of *Δ***σ** is reliably reconstructed by *Δ*σ^*^ in Fig. 4(e). The comparison in Fig. 4 indicates that the conductivity reconstructed based on the OO-SME model has a higher accuracy than the existing models.

**Fig. 5:**
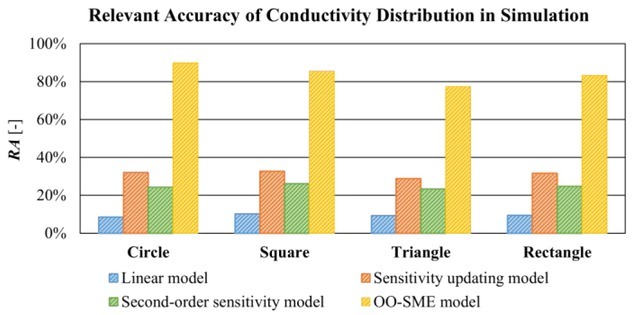
Comparison of *RA* of reconstructed conductivity based on the linear model, sensitivity updating model, second-order sensitivity model, and OO-SME model in the simulation.

Fig. 5 shows *RA* of reconstructed conductivity *Δ*σ^*^ in Fig. 4. On average, *RA* with the linear model is 9.39%. *RA* with the OO-SME model is 83.98%. *RA* with the sensitivity updating model is 31.30% and *RA* with the second-order sensitivity model is 24.61%. Compared to the linear model, *RA* with the sensitivity updating model and the second-order sensitivity model increased slightly by 21.91% and 15.24% respectively. *RA* with the OO-SME model increased significantly by 73.59%. The comparisons in Fig. 5 show that the reconstructed conductivity *Δ*σ^*^ with the OO-SME model has a higher accuracy to evaluate the ideal conductivity change *Δ***σ** than the existing models.

## Conductivity reconstruction by experiment

### Experimental setup

**Fig. 6:**
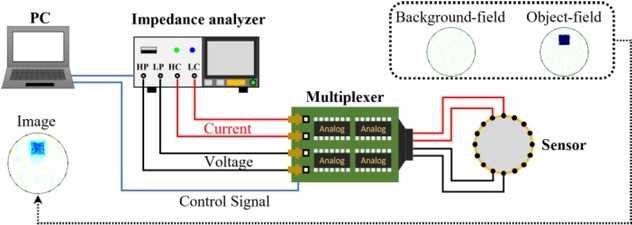
Experimental setup of EIT system

Fig. 6 shows the experimental setup of an EIT system, which consists of 4 parts, a personal computer (PC), an impedance analyzer, a digital multiplexer, and an EIT sensor. The impedance analyzer is IM3570 made by Hioki. The multiplexer is made based on Arduino, which has 16 channels to switch on and off between different electrode-combinations for current-stimulation and voltage-measurement. The sensor is a polylactic acid-made circular tank printed with a 3D printer. The diameter of the tank is *d* = 100 mm. The 16 electrodes made of stainless screw are mounted along the circumference of the tank evenly.

### Experimental method

As shown in Fig. 6, the PC controls the signals to trigger the impedance analyzer and switch on and off the channels on the multiplexer. The impedance analyzer generates a current signal on two output channels (HC and LC) to stimulate the target and measures the voltage signal from the target via two input channels (HP and LP), from which the impedance of the target is calculated. The multiplexer chooses 4 of 16 channels to stimulate the current and measure the voltage, the electrode-combinations for current-stimulation and voltage-measurement in the experiment are the same as in the simulation.

The experiment is conducted as follow. At first, the impedance from the background-field and the object-field are measured. Then, the voltages of background-field **U**^b^^*^ and object-field **U**^o^^*^ are extracted, and the voltage change ΔU^*^ from **U**^b^^*^ to **U**^o^^*^ is calculated. At last, Δσ^*^ is reconstructed by matching ΔU^*^ with ***u***(Δ**σ**) based on different conductivity reconstruction models.

### Experimental condition

In the experiment, the meat sample from pig rump was used. The background-field is fat. The object-field is a lean meat mass enclosed by fat. The conductivity of fat and lean meat are *σ^f^* = 0.021 S/m and *σ^m^* = 0.267 S/m respectively at *f* = 100 Hz [29]. The lean meat masses with different shapes and sizes are measured, which have the same dimension as the objects in the simulation. The amplitude of current stimulation is *I* = 1mA. The current frequency is *f* = 100 Hz.

### Experimental results

**Fig. 7:**
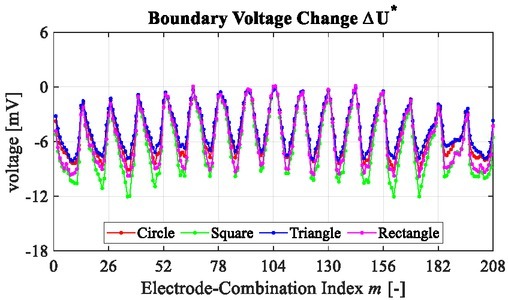
Voltage changes of different objects in the experiment.

Fig. 7 shows the voltage change ΔU^*^ of different lean meat masses in the experiment. ΔU^*^ is divided into 16 loops, and in each loop it has 13 measurements, where the value varies periodically with the changing of electrode-combinations. Fig. 7 indicates that the conductivity change in the meat sample is able to be measured reliably by the voltage change on the boundary.

**Fig. 8:**
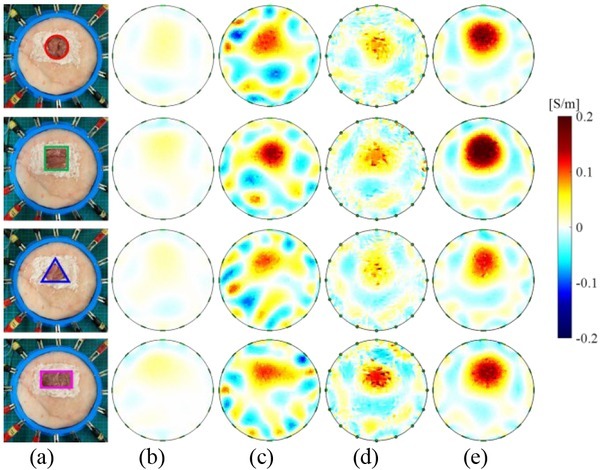
Reconstructed conductivity based on different conductivity reconstruction models in the experiment. (a) Object-fields; (b) Linear model; (c) Sensitivity updating model; (d) Second-order sensitivity model; (e) OO-SME model.

Fig. 8 shows the reconstructed conductivity *Δ*σ^*^ of lean meat mass based on the linear model, two nonlinear models, and the OO-SME model. Fig. 8(a) shows four object-fields of lean meat masses. Fig. 8(b), (c), and (d) show *Δ*σ^*^ based on the linear model, sensitivity updating model, and second-order sensitivity model, respectively. Fig. 8(e) shows *Δ*σ^*^ based on the OO-SME model. Compared to the lean meat masses in Fig. 8(a), *Δ*σ^*^ in Fig. 8(b), (c) and (d) display the position of lean meat mass only, in which the quantitative value of *Δ*σ^*^ is not accurate. In contrast, reconstructed *Δ*σ^*^ in Fig. 8(e) has a high agreement with ideal *Δ***σ**. The comparison in Fig. 8 indicates that the proposed OO-SME model reconstructs the lean meat mass in meat sample accurately. Furthermore, the reconstruction can be used to evaluate the mass of lean meat quantitatively.

**Fig. 9:**
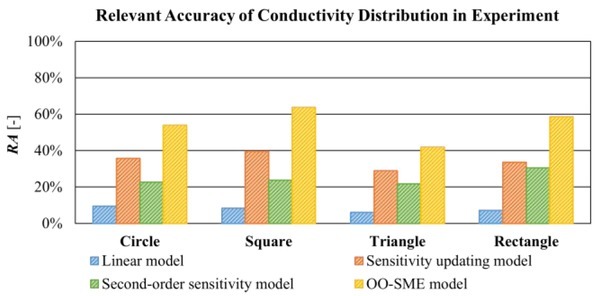
Comparison of *RA* of reconstructed conductivity based on the linear model, sensitivity updating model, second-order sensitivity model, and OO-SME model in the experiment.

Fig. 9 shows *RA* of reconstructed conductivity *Δ*σ^*^ in Fig. 8. On average, *RA* with the linear model is 7.74%. *RA* with the OO-SME model is 54.60%. *RA* with the sensitivity updating model is 34.45% and *RA* with the second-order sensitivity model is 24.62%. Compared to the linear model, *RA* with the sensitivity updating model and the second-order sensitivity model increased slightly by 26.71% and 16.88% respectively. *RA* with the OO-SME model increased significantly by 46.86%. In conclusion, images in EIT reconstructed based on the OO-SME model have a higher accuracy to quantify the conductivity change in lean meat mass.

## Discussion

### Approximation error of the OO-SME model

**Fig. 10:**
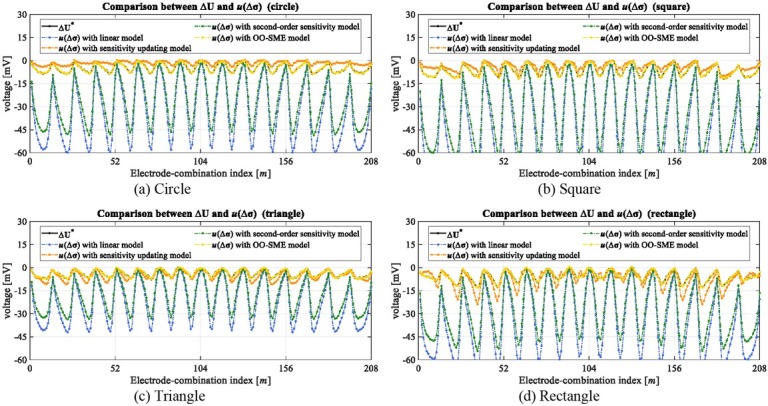
Comparison between ΔU^*^ and *u*(Δσ) based on different conductivity reconstruction models in the simulation.

Due to the approximation error ***e*** between ΔU^*^ and ***u***(Δ**σ**), reconstructed conductivity Δσ^*^ has low accuracy. Fig. 10 shows the comparison between ΔU^*^ and ***u***(Δ**σ**) from different conductivity reconstruction models in the simulation. Fig. 10(a), (b), (c) and (d) are corresponding to four different objects, respectively. The comparison shows that ***e*** from the linear model and the second-order sensitivity model are obvious. The relative ratio of ***e*** to ΔU^*^ reaches up to 622% in the linear model and 477% in the second-order sensitivity model on average. Comparing to the linear model and second-order sensitivity model, even though ***e*** is significantly reduced in the sensitivity updating model, the relative ratio of ***e*** is still around 50% on average. In contrast, ***e*** from the OO-SME model is sufficiently eliminated, the relative ratio of ***e*** is as low as 2.4% on average. The comparison in Fig. 10 indicates that non-negligible approximation error ***e*** in the existing models is significantly reduced in the OO-SME model.

The approximation error ***e*** is caused by inaccurate sensitivity matrix estimation in different conductivity reconstruction models. In the linear model for approximation of ***u***(Δ**σ**) as (7), the simplification of omitting the change of ***f*** and replacing **J***^b^* with **S***^b^* leads to non-ignorable ***e***. Consequently, Δσ^*^ of different objects in Fig. 4(b) have similar distributions, and the magnitude of Δσ^*^ does not match with Δ**σ**. In the sensitivity updating model for approximation of ***u***(Δ**σ**) as (20), ***e*** is expected to be reduced by replacing **S***^b^* with **S**^b^^*^. Even though **S**^b^^*^ from **σ**^b^^*^ has a higher accuracy compared to **S***^b^*, it is still not accurate enough to reduce ***e*** obviously. Therefore, as shown in Fig. 4(c), the accuracy of Δσ^*^ is slightly improved. In the second-order sensitivity model for approximation of ***u***(Δ**σ**) as (21), ***e*** is expected to be reduced by considering the estimated second-order sensitivity **S***^b^*^†^. Since **S***^b^*^†^ is much smaller than **S***^b^*, leaving the contribution of **S***^b^*^†^ on ***u***(Δ**σ**) is limited. Thus, ***e*** is slightly reduced compared to the linear model. As a result, as shown in Fig. 4(d), the accuracy of Δσ^*^ has not been improved obviously. However, different from the derivation in the linear model based on the Taylor formula, ***u***(Δ**σ**) in the OO-SME model is derived as (2) based on the divergence theorem, in which the influence of Δ**S** on ***u***(Δ**σ**) is completely considered. Therefore, ***e*** is eliminated in the OO-SME model and the accuracy of Δσ^*^ is significantly improved compared to the existing models. As shown in Fig. 4(e), Δσ^*^ based on the OO-SME model obtained the object shape and size accurately.

### Contribution of sensitivity change Δ**S** to **u**(Δ**σ**)

**Fig. 11:**
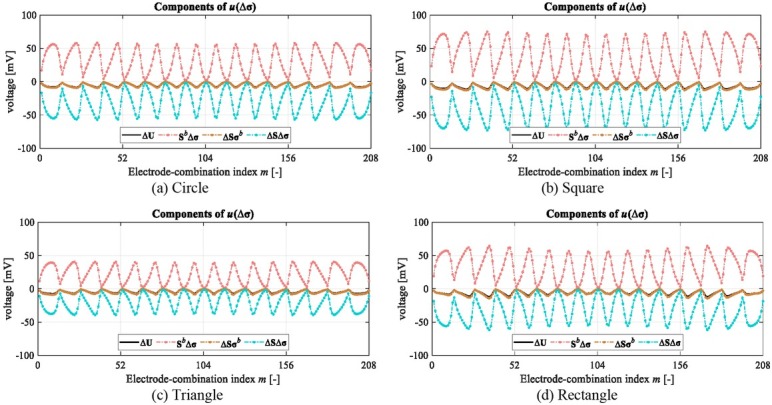
Comparison of components of *u*(Δσ) with different objects in the simulation.

As expressed by (2), ***u***(Δ**σ**) based on the OO-SME model contains three components, all of which have non-ignorable contributions to ***u***(Δ**σ**). Fig. 11 shows the three components of ***u***(Δ**σ**) from different objects in the simulation. On average, the ratio of **S***^b^*Δ**σ** to Δ**U** is around -6.34, the ratio of Δ**Sσ***^b^* to Δ**U** is around 1.18, and the ratio of Δ**S**Δ**σ** to Δ**U** is around 6.16. The comparison implies two facts. Firstly, the nonlinear operator ***f*** acting on **σ** changes from ***f****^b^* to ***f****^o^* with **σ** changes from **σ***^b^* to **σ***^o^*; the contribution of change of ***f*** to ***u***(Δ**σ**) should be considered by sensitivity matrix change Δ**S**. Secondly, the contribution of Δ**S** to ***u***(Δ**σ**) is expressed as Δ**S**(**σ***^b^*+Δ**σ**), which consists of two parts; one is Δ**S**Δ**σ** implying that Δ**S** induced by Δ**σ** influences the operator ***f*** acting on Δ**σ**; another is Δ**Sσ***^b^* implying that the influence of Δ**S** on **σ***^b^* has to be considered to approximate ***u***(Δ**σ**).

Omitting the change of ***f*** in the linear model led to a non-negligible ***e***. The reduction of ***e*** from the optimized sensitivity matrix **S**^b^^*^ or **S***^b^* + **S***^b^*^†^ in the two nonlinear models is limited since the contribution of Δ**S** corresponding to the change of ***f*** is not properly considered. In the OO-SME model, ***e*** is eliminated by completely considering the contribution of Δ**S** to ***u***(Δ**σ**), including the components relevant to **σ***^b^* and Δ**σ** simultaneously.

### Comparison of sensitivity matrices

**Fig. 12:**
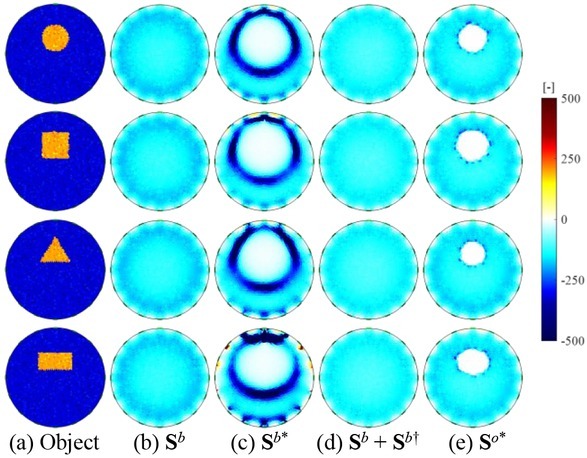
Comparison of sensitivity based on different conductivity reconstruction models in the simulation. (a) Object-field; (b) S*^b^* in linear model; (c) S^b^^*^ in sensitivity updating model; (d) S*^b^* + S*^b^*^†^ in second-order sensitivity model; (e) S^o^^*^ in OO-SME model.

Fig. 12 shows the comparison of sensitivity matrix with different conductivity reconstruction models in the simulation, where the sensitivity of each element from all electrode-combinations are collected. Fig. 12(a) shows the conductivity of object-fields. Fig. 12(b), (c) and (d) show **S***^b^* in the linear model, **S**^b^^*^ in the sensitivity updating model and **S***^b^* + **S***^b^*^†^ in the second-order sensitivity model, respectively. Fig. 12(e) shows **S**^o^^*^ in the OO-SME model. The comparison indicates that the sensitivity matrix change Δ**S** is necessary to be considered. Compared to the **S***^b^* from a homogeneous background-field and the **S***^b^* + **S***^b^*^†^ that is optimized from **S***^b^* directly, **S**^b^^*^ contains information of the object but it is not accurate. In contrast, **S**^o^^*^ has high accuracy, from which the object information is reliably detected. The comparison in Fig. 12 indicates that the accuracy of the sensitivity matrix in the OO-SME model is significantly improved, from which the estimated sensitivity matrix change ΔS^*^ is calculated to optimize ***u***(Δ**σ**).

**Fig. 13:**
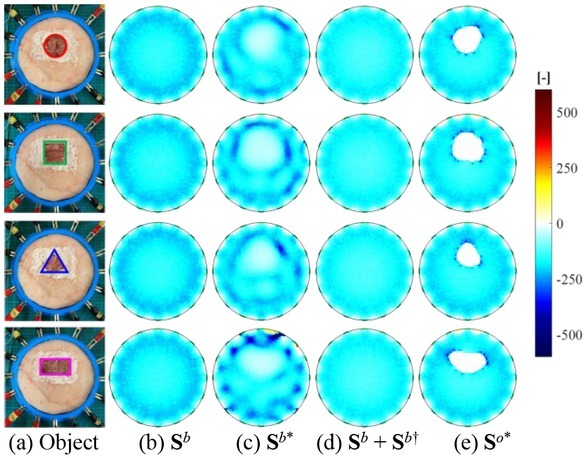
Comparison of sensitivity based on different conductivity reconstruction models in the experiment. (a) Object-field; (b) S*^b^* in linear model; (c) S^b^^*^ in sensitivity updating model; (d) S*^b^* + S*^b^*^†^ in second-order sensitivity model; (e) S^o^^*^ in OO-SME model.

Fig. 13 shows the comparison of the sensitivity matrix in the experiment, where the sensitivity of each element from all electrode-combinations are collected. Fig. 13(a) shows the object-field. Fig. 13(b), (c), and (d) show **S***^b^* in the linear model, **S**^b^^*^ in the sensitivity updating model and **S***^b^* + **S***^b^*^†^ in the second-order sensitivity model respectively. Fig. 13(e) shows **S**^o^^*^ in the OO-SME model. Similar as in Fig. 12, **S**^o^^*^ in the OO-SME model has a higher accuracy compared to the existing models, from which ΔS^*^ is estimated accurately to optimize ***u***(Δ**σ**).

### Quantitative evaluation of measurement object by the OO-SME model

The conductivity reconstruction based on the OO-SME model has high accuracy to evaluate the measurement object quantitatively, which improves the reliability of EIT application in the biomedical field, such as evaluation of effect of EMS on muscle compartments. In this study, the lean meat mass enclosed by fat is accurately reconstructed by the proposed OO-SME model, the relative accuracy *RA* reaches up 83.98% in the simulation and 54.60% in the experiment, respectively. Compared to the *RA* of 34.45% in the simulation and 31.30% in the experiment from the sensitivity updating model, the reliability of reconstruction from the OO-SME model is significantly increased. Thus, the OO-SME model can be used for quantitative evaluation of measurement objects in the biomedical fields of EIT applications.

## Conclusions

1. The approximation error in the OO-SME model proposed in this study is eliminated compared to the existing models. The reconstructed conductivity from the OO-SME model has higher accuracy to reflect the shape and size of measurement object.
2. The lean meat mass in meat sample is accurately reconstructed by the OO-SME model, from which the lean meat mass could be quantitative evaluated.
3. The relative accuracy of lean meat mass from the reconstructed conductivity based on the OO-SME model reaches up to 83.98% in the simulation and 54.60% in the experiment. The reconstruction has a higher reliability to evaluate the lean meat mass quantitatively.
